# Clinical perspectives on the value of testing for *STK11* and *KEAP1* mutations in advanced NSCLC

**DOI:** 10.3389/fonc.2024.1459737

**Published:** 2024-12-05

**Authors:** Michelle Shiller, Melissa Johnson, Robert Auber, Sandip Pravin Patel

**Affiliations:** ^1^ Department of Pathology, Baylor University Medical Center, Dallas, TX, United States; ^2^ Department of Medical Oncology, Sarah Cannon Cancer Research Institute, Nashville, TN, United States; ^3^ Department of Molecular Oncology, PathGroup, Nashville, TN, United States; ^4^ Division of Hematology and Oncology, Moores Cancer Center, University of California, San Diego, San Diego, CA, United States

**Keywords:** protein serine-threonine kinases, kelch-like ECH-associated protein 1, c proto-oncogene proteins p21(ras), carcinoma, non-small-cell lung, immunotherapy

## Abstract

Standard first-line therapy for patients with metastatic non-small cell lung cancer (mNSCLC) without identified actionable mutations consists of regimens comprising immune checkpoint inhibitors (ICIs), alone or in combination with platinum-based chemotherapy (CTx). However, approximately 20–30% of patients with mNSCLC (including some patients with high tumor programmed cell death ligand-1 expression) display primary resistance to ICIs, either alone or in combination with CTx. Mutations in tumor suppressor genes *serine/threonine kinase 11* (*STK11*), and *Kelch-like ECH-associated protein 1* (*KEAP1*) often detected in patients with *Kirsten rat sarcoma virus* mutations, are associated with an aggressive disease phenotype and resistance to standard ICI regimens. Consequently, there is an important need for effective treatments for patients with NSCLC with *STK11* or *KEAP1* mutations. In this article, we describe new data on the prevalence of *STK11* and *KEAP1* mutations in a large clinical population, consider practicalities around the detection of these mutations using available biomarker testing methodologies, and describe experiences of managing some of these difficult-to-treat patients in our clinical practice.

## Introduction

Globally, lung cancer is the second most commonly diagnosed malignancy, but the leading cause of cancer mortality (1). Among the more than 2 million cases of lung cancer diagnosed each year (1), most are diagnosed at an advanced stage, leading to a poor prognosis for these patients (2). The most common lung cancer subtype, non-small cell lung cancer (NSCLC) accounts for 85% of cases (3), but can be further divided by histological subtype, with adenocarcinoma being the most common (4, 5). NSCLC is driven by an array of genomic events and molecular mechanisms which lead to disparities in outcomes (5, 6). As our understanding of the molecular pathology of NSCLC has advanced, so has our ability to distinguish between specific molecular subtypes, to reach more accurate judgments regarding the prognoses of the patients in our care, and to tailor treatment regimens that target the specific etiology of each individual’s disease.

Advances in molecular subtyping have facilitated the development of personalized treatments for NSCLC (7), and organizations such as the National Comprehensive Cancer Network^®^ (NCCN^®^), the American Society of Clinical Oncology (ASCO), and the European Society for Medical Oncology (ESMO) now advocate the use of biomarker testing at the time of diagnosis of advanced/metastatic NSCLC, to ensure that patients with actionable molecular alterations are treated with the appropriate targeted therapies (8–10). For those without actionable mutations, the addition of an anti-programmed cell death ligand-1 (PD-L1)/programmed cell death-1 (PD-1) agent to chemotherapy (CTx) provides a survival benefit compared with chemotherapy alone (11–18). NCCN Clinical Practice Guidelines in Oncology (NCCN Guidelines®) recommend the use of chemoimmunotherapy (CIT) regimens as options in this broad subset of patients, irrespective of PD-(L)1 expression status (9). However, in some patients with mutations in certain tumor suppressor genes, including the *serine/threonine kinase 11*(*STK11*) and *Kelch-like ECH-associated protein* (*KEAP1*) genes, the benefit of CIT appears to be less clear (19–24). Understanding the molecular and cellular basis of resistance to PD-(L)1 inhibition may allow us to better refine treatment strategies for patients with *STK11* and *KEAP1* mutation-positive NSCLC. In this article we consider the latest information and provide our perspectives on the treatment of patients with NSCLC tumors harboring *STK11* and *KEAP1* mutations.

## Therapeutic options for patients with advanced NSCLC and *KRAS* mutations

The *Kirsten rat sarcoma virus* (*KRAS*) mutation is the most frequently occurring genomic abnormality in NSCLC, being present in up to 30% of tumors (19). The KRAS inhibitors sotorasib and adagrasib were approved by the US Food and Drug Administration (FDA) for second-line treatment of *KRAS* G12C-mutated NSCLC (25, 26) after demonstrating acceptable objective response rates in these patients after failure of standard therapies, including CIT (27, 28). More information is required on the effectiveness of KRAS inhibitors for first-line treatment of metastatic (m)NSCLC; the results of several ongoing trials of KRAS inhibitors in combination with PD-(L)1 inhibitors are awaited.

In the absence of data on first-line therapy for tumors with *KRAS* mutations (of any type), the NCCN Guidelines^®^ continue to recommend the use of CIT as a treatment option (9). Although immunotherapy (IO), with or without CTx, has demonstrated efficacy in patients with a *KRAS* mutation (24, 29), there are notable variabilities in reported outcomes among these patients, some of which may be explained by genetic heterogeneity. Firstly, while *KRAS* mutations arise most commonly in the G12 codon (30, 31), increasing evidence suggests that there are important differences between the three most common G12 mutations: G12C (40% of *KRAS* mutations in lung), G12V (22%), and G12D (16%) (32). G12C and G12V mutations both result from a smoking-induced point mutation, whereas G12D mostly occurs in low/never-smokers (33, 34). G12C and G12V appear to be induced by chronic exposure to tobacco carcinogens, which also increases tumor mutational burden (TMB) and PD-1/PD-L1 expression, both of which are predictors of good response to immunotherapy (35–39). In contrast, neither PD-L1 expression nor TMB are enriched in tumors with *KRAS* G12D mutations. Rather, such tumors have an immunologically ‘cold’ immune microenvironment, with relatively low expression of tumor neoantigens and little T-cell infiltration (39). Consequently, compared with the favorable outcomes observed in patients with tumors bearing *KRAS* G12C or G12V mutations, those with G12D mutations have worse outcomes on any line of anti-PD-(L)1 therapy (33, 40).

Other forms of genomic diversity exist among patients with NSCLC tumors harboring *KRAS* mutations. As described in the following sections, recent advances in our understanding of the influence of this genomic diversity are beginning to inform our clinical practice.

## The influence of *STK11* and *KEAP1* mutations on outcomes of advanced NSCLC

Around half of patients with identified *KRAS* mutations have been found to harbor additional cancer-associated mutations, most frequently in tumor suppressor genes, specifically *STK11* and *KEAP1* (41–43). These genes normally act to inhibit cell proliferation and tumor development, so their loss or inactivation removes this inhibition of cell proliferation and contributes to abnormal proliferation of the tumor cells (42–44). Tumors bearing such mutations tend to have an aggressive disease biology, due to alterations in metabolic pathways that contribute to an immunosuppressive tumor environment (24, 45).

As mentioned, the results of several analyses suggest that patients with *KRAS* mutations benefit from standard-of-care CIT (24, 29, 46); however, the presence of concurrent *STK11* and *KEAP1* may lead to relatively poor outcomes (19, 20, 22, 47–49). In some real-world studies, overall survival (OS) outcomes in patients with advanced NSCLC treated with CTx, IO, or CIT were found to be worse among patients with *KRAS*-mutated NSCLC with co-mutations in *STK11* or *KEAP1*, compared with tumors with *KRAS* mutations alone (**Table 1A**).

**Table 1:** Impact of *STK11*, *KEAP1*, and *KRAS* mutations or co-mutations on survival outcomes with standard therapies (A, B), and on survival outcomes with IO + CTx combinations vs. CTx alone (C).

A. Patients with *KRAS* mutations: HRs for PFS and OS between subgroups with/without *STK11* and/or *KEAP1* co-mutations			
Analysis	Therapy type	PFS HR (95% CI)^a^	OS HR (95% CI)^a^
HRs for *KRAS*m *+ STK11*m vs. *KRAS*m *+ STK11*wt			
**Julian^b^ (** 49)	IO ± CTx, or CTx (1L)	–	1.00 (0.67, 1.51) P=0.99
**Julian^b^ (** 49)	IO ± CTx, or CTx (2L)	–	1.02 (0.56, 1.84) P=0.96
**Peters (** 48)	IO ± CTx, or IO + CTx + VEGFi	–	1.63 (1.12, 2.39) P=0.0136
HRs for *KRAS* G12Cm + *KEAP1*m vs. *KRAS* G12Cm + *KEAP1*wt			
**Julian (** 49)	IO ± CTx, or CTx (1L)	–	1.57 (0.95, 2.60) P=0.08
**Julian (** 49)	IO ± CTx, or CTx (2L)	–	1.63 (0.77, 3.45) P=0.20
HRs for *KRAS* G12Cm + *STK11*m + *KEAP1*m vs. *KRAS* G12Cm + *STK11*wt + *KEAP1*wt			
**Julian (** 49)	IO ± CTx, or CTx (1L)	–	1.93 (1.35, 2.75) P<0.001
**Julian (** 49)	IO ± CTx, or CTx (2L)	–	2.20 (1.27, 3.81) P=0.005

B. HRs for PFS and OS between subgroups with/without *STK11*, *KEAP1* and *STK11* + *KEAP1* mutations			
Analysis	Therapy type	PFS HR (95% CI)^c^	OS HR (95% CI)^c^
HRs for *STK11*m vs. *STK11*wt			
**Papillon-Cavanagh (** 22)	CTx	1.32 (1.04, 1.68) P=0.01–0.05	1.19 (0.89, 1.6)^d^
**Papillon-Cavanagh (** 22)	Anti PD-(L)1	1.33 (0.93, 1.9)^d^	1.43 (0.91, 2.26)^d^
**Cordeiro de Lima (** 51)	Anti PD-(L)1	1.31 (1.12, 1.88) P=0.02	1.33 (1.13, 2.21) P=0.001
**Julian (** 49)	IO ± CTx, or CTx (1L)	-	1.27 (1.00, 1.62) P=0.05
**Julian (** 49)	IO ± CTx, or CTx (2L)	–	1.32 (0.95, 1.83) P=0.10
**Peters (** 48)	IO ± CTx, or IO + CTx + VEGFi^e^	–	1.46 (1.12, 1.92) P=0.0070
HRs for *KEAP1*m vs. *KEAP1*wt			
**Papillon-Cavanagh (** 22)	CTx	1.53 (1.22, 1.93) P ≤ 0.001	1.49 (1.14, 1.95) P=0.001–0.01
**Papillon-Cavanagh (** 22)	Anti PD-(L)1	1.71 (1.2, 2.45) P=0.001–0.01	1.71 (1.04, 2.81) P=0.01–0.05
**Cordeiro de Lima (** 51)	Anti PD-(L)1	1.27 (0.97, 1.29) P=0.32	1.19 (1.18, 1.31) P=0.04
**Julian (** 49)	IO ± CTx, or CTx (1L)	–	1.21 (1.00, 1.48) P=0.06
**Julian (** 49)	IO ± CTx, or CTx (2L)	–	1.20 (0.93, 1.55) P=0.17
**Peters (** 48)	IO ± CTx, or IO + CTx + VEGFi^e^	–	1.26 (0.95, 1.68) P=0.1172
HRs for *STK11*m + *KEAP1*m vs. *STK11*wt + *KEAP1*wt			
**Julian (** 49)	IO ± CTx, or CTx (1L)	–	1.81 (1.44, 2.26) P<0.001
**Julian (** 49)	IO ± CTx, or CTx (2L)	–	1.83 (1.35, 2.49) P<0.001

C. HRs for PFS and OS between investigational therapy and CTx (for subgroups with/without *KRAS, STK11* and *KEAP1* mutations)					
Analysis	Comparison	PFS HR (95% CI)^f^		OS HR (95% CI)^f^	
*KRAS* subgroups		*KRAS*m	*KRAS*wt	*KRAS*m	*KRAS*wt
**POSEIDON^g^**	Anti-PD-L1 vs. CTx	0.82 (0.53, 1.29) (57)	NR	0.74 (0.50, 1.09) (70)	0.87 (0.68, 1.12) (70)
**CheckMate 227 (** 54)	Anti-PD-1 + Anti-CTLA-4 vs. CTx	–		0.79 (0.55, 1.12)	0.73 (0.56, 0.95)
**CheckMate 9LA (** 55)	Anti-PD-1 + Anti-CTLA-4 + CTx vs. CTx	0.74 (0.50, 1.10)	0.73 (0.52, 1.02)	0.72 (0.48, 1.08)	1.0 (0.72, 1.39)
**POSEIDON^g^**	Anti-PD-1 + Anti-CTLA-4 + CTx vs. CTx	0.57 (0.35, 0.92) (57)	NR	0.55 (0.36, 0.83) (70)	0.78 (0.61, 1.00) (70)
*STK11* subgroups		*STK11*m	*STK11*wt	*STK11*m	*STK11*wt
**POSEIDON^g^**	Anti-PD-L1 vs. CTx	1.02 (0.55, 1.93) (57)	NR	1.02 (0.59, 1.80) (70)	0.79 (0.63, 1.00) (70)
**CheckMate 227 (** 54)	Anti-PD-1 + Anti-CTLA-4 vs. CTx	–	–	0.78 (0.48, 1.27)	0.75 (0.59, 0.94)
**CheckMate 9LA (** 55)	Anti-PD-1 + Anti-CTLA-4 + CTx vs. CTx	0.61 (0.37, 1.00)	0.77 (0.57, 1.04)	0.79 (0.48, 1.28)	0.90 (0.67, 1.22)
**POSEIDON^g^**	Anti-PD-1 + Anti-CTLA-4 + CTx vs. CTx	0.47 (0.23, 0.93)	NR	0.57 (0.32, 1.04) (70)	0.71 (0.56, 0.90) (70)
*KEAP1* subgroups		*KEAP1*m	*KEAP1*wt	*KEAP1*m	*KEAP1*wt
**POSEIDON^g^**	Anti-PD-L1 vs. CTx	1.51 (0.55, 5.25) (57)	NR	0.77 (0.31, 2.15) (70)	0.83 (0.70, 0.98) (70)
**CheckMate 227 (** 54)	Anti-PD-1 + Anti-CTLA-4 vs. CTx	–	–	0.31 (0.14, 0.70)	0.80 (0.65, 1.00)
**CheckMate 9LA (** 55)	Anti-PD-1 + Anti-CTLA-4 + CTx vs. CTx	0.34 (0.14, 0.83)	0.79 (0.60,1.03)	0.51 (0.24, 1.08)	0.94 (0.71, 1.23)
**POSEIDON^g^**	Anti-PD-1 + Anti-CTLA-4 + CTx vs. CTx	0.94 (0.33, 3.35) (57)	NR	0.43 (0.16, 1.25) (70)	0.76 (0.64, 0.90) (70)

^a^HR >1.0 indicates that the comparison favors the subgroup without *STK11*/*KEAP1* co-mutations; ^b^ *KRAS* G12Cm only; ^c^HR >1.0 indicates that the comparison favors the subgroup without *STK11*/*KEAP1* mutations; ^d^P value not available; ^e^Patients received pembrolizumab (n=94), pembrolizumab + chemotherapy, NSQ (n=462), pembrolizumab + chemotherapy, SQ (n=122), or atezolizumab + bevacizumab + chemotherapy (n=4); ^f^HR <1.0 indicates that the comparison favors the investigational therapy; ^g^Analysis performed in patients with NSQ histology only.

1L, first-line; 2L, second-line; CI, confidence interval; CTLA-4, cytotoxic T−lymphocyte-associated antigen 4; CTx, chemotherapy; HR, hazard ratio; IO, immunotherapy; KEAP1(m/wt), Kelch-like ECH-associated protein 1 (mutation-positive/wild-type); KRAS(m/wt), Kirsten rat sarcoma virus (mutation-positive/wild-type); NR, not reached; NSQ, non-squamous; OS, overall survival; PD-(L)1; programmed cell death (ligand)-1; PFS, progression-free survival; SQ, squamous; STK11(m/wt), serine/threonine kinase 11 (mutation-positive/wild-type); VEGF, vascular endothelial growth factor.

The presence of *STK11* and/or *KEAP1* mutations (without concurrent *KRAS* mutations) also appears to predict for poor therapeutic outcomes in patients with advanced NSCLC. In some studies, OS and progression-free survival (PFS) were worse among patients with *STK11* and/or *KEAP1* mutations treated with CTx, IO, or CIT, compared with patients with *STK11* or *KEAP1* wild-type tumors (**Table 1B**). *KEAP1* mutations have also been shown to confer resistance to radiotherapy (50). Although it is currently recommended that patients with *STK11* or *KEAP* mutation-positive NSCLC should receive standard-of-care CIT (8–10), the introduction of IO has not improved the outlook for these patients (22, 51), who continue to have relatively poor outcomes, irrespective of the treatment regimen given (**Table 1B**). There is no firm consensus on whether the presence of these mutations is negatively prognostic or predicts poor survival outcomes with specific therapies.

There is a clear unmet need for effective treatments for patients with *STK11* and *KEAP1* mutations (with or without concurrent *KRAS* mutations). Select treatment regimens comprising PD-(L)1 inhibitors in combination with CTx, and specific combinations incorporating anti-cytotoxic T−lymphocyte-associated antigen 4 (CTLA-4) agents are NCCN-recommended and FDA-approved first-line treatment options for patients with mNSCLC with performance status (PS) 0–1 and without driver mutations (9, 52, 53). In three Phase 3 studies (CheckMate 227 [NCT02477826], POSEIDON [NCT03164616] and CheckMate 9LA [NCT03215706]), conducted in treatment-naïve patients with *EGFR* and *ALK* wild-type mNSCLC, dual PD-(L)1 and CTLA-4 inhibition with CTx led to improvements in OS and PFS compared with CTx alone (54–56). Exploratory analyses suggest that such regimens may also be beneficial for patient subgroups with historically poor outcomes (specifically, patients with *STK11* and/or *KEAP1* mutations). In each of these studies, patients treated with these regimens achieved improvements in OS compared to those treated with CTx, in subsets both with and without *STK11*, *KEAP1*, or *KRAS* mutations (**Table 1C**) (54, 55, 57). However, considering the small numbers in these subsets, prospective analyses based on larger sample sizes are required to adequately evaluate the effects of these regimens in these difficult-to-treat patients, and to fully establish the value of *STK11*, *KEAP1*, and *KRAS* mutations as biomarkers to inform routine clinical practice.

## Prevalence of *STK11* and *KEAP1* mutations in NSCLC: pathological subtyping of NSCLC in a large clinical population

At Baylor University Medical Center, Dallas, Texas patients with NSCLC undergo routine pathological subtyping using the OncoKB platform. Subsequently, biomarker testing is conducted at PathGroup [RA] using the Endeavor test, a broad panel designed to detect variants in more than 500 cancer genes, with full exon coverage, as well as TMB.

The authors [MS and RA] analyzed tumor samples collected from 3,745 patients with NSCLC of any stage, including patients with actionable oncogenic alterations. Patients with mixed histology and unknown PD-L1 status were excluded from relevant subgroup analyses. In addition to routine biomarker testing using the Endeavor test, the PGDx elio™ tissue complete assay was used to evaluate TMB; the results were cross-validated with FoundationOne^®^ CDx and MSK-IMPACT^®^ assays. TMB-high status was defined as ≥16.0 mutations/megabase (mut/Mb), TMB-low status was defined as <16.0 mut/Mb. PD-L1 testing was performed using the PD-L1 IHC 22C3 pharmDx assay; a tumor proportion score (TPS) ≥1% was deemed to be positive and TPS <1% negative (indeterminate results were excluded from the analysis).

The current analysis revealed that *KRAS* mutations were present in 27.5% of NSCLC tumors, similar to previous reports that indicate the overall prevalence to be ~30% (19). In this analysis, we report the prevalence of pathogenetic variants only. Without this curation, our dataset could include ‘passenger’ mutations that would unknowingly be interpreted as false positives for oncogenicity. Other studies may not have curated their prevalence data in the same way. Accordingly, in the current analysis, *STK11* mutations and *KEAP1* mutations were identified relatively infrequently (in 8.6% and 5.7% of patients, respectively), compared with the previously reported prevalence ranges of 18–25% for *STK11* (24, 58, 59) and 10–15% for *KEAP1* (24, 59). *KRAS* was co-mutated with *STK11* in 4.0% of tumors, and with *KEAP1* in 1.9% of tumors. Co-mutations in *KEAP1* and *STK11* occurred in 1.5%, while 0.8% harbored triple mutations in *KRAS*, *STK11*, and *KEAP1*.

## Relationships between mutation frequencies and tumor histology, TMB, and PD-L1 status

**Figure 1:**
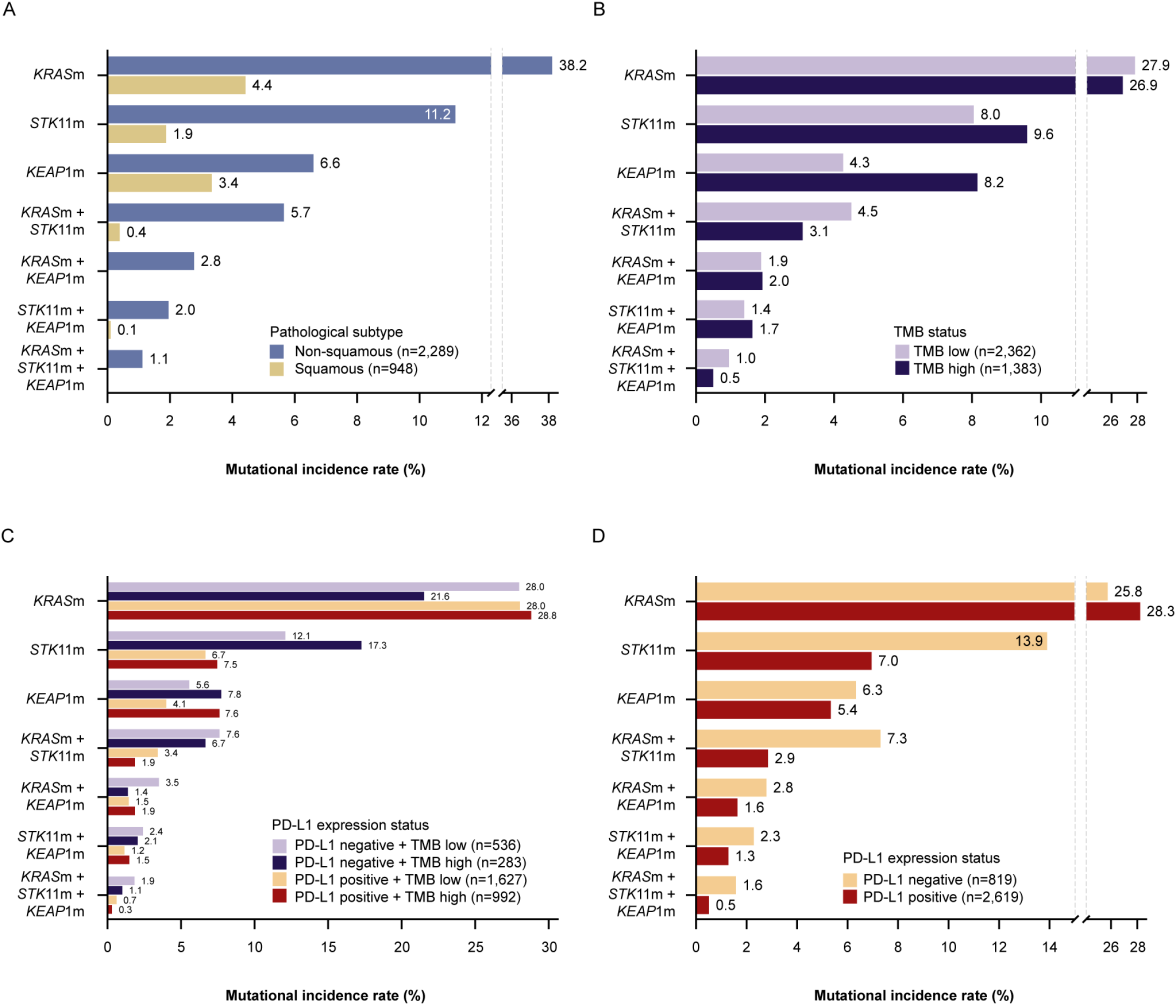
Incidence of *KRAS*, *STK11*, and *KEAP1* (co-)mutations by pathological subtype **(A)**, TMB^a^ status **(B)**, TMB^a^ and PD-L1 expression^b^ status **(C)**, and PD-L1 expression^b^ status **(D)**. ^a^The PGDx elio™ tissue complete assay was used to determine TMB high (≥16.0 mut/Mb) vs. TMB low (<16.0 mut/Mb) status; ^b^The PD-L1 IHC 22C3 pharmDx assay was used to determine PD-L1 positive (TPS ≥1%) vs. PD-L1 negative (TPS <1%) status. KEAP1(m), Kelch-like ECH-associated protein 1 (mutation-positive); KRAS(m), Kirsten rat sarcoma virus (mutation-positive); PD-L1, programmed cell death ligand-1; STK11(m), serine/threonine kinase 11 (mutation-positive); TMB, tumor mutational burden; TPS, tumor proportion score.

All the mutations and co-mutations evaluated (in *STK11, KEAP1*, and *KRAS)* were markedly more frequent in tumors with non-squamous (n=2,289) than squamous histology (n=948) (**Figure 1A**). Moreover, no squamous cell tumors harbored *KRAS* and *KEAP1* co-mutations or triple mutations (in *KRAS*, *STK11* and *KEAP1)*. The marked differences between patients with non-squamous and squamous histology were also apparent in subgroups defined both by histology and either TMB (**Supplementary Figure S1A**) or PD-L1 expression status (**Supplementary Figure S1B**).

The curation of prevalence data to identify only pathogenic variants of *KRAS*, *STK11*, and *KEAP1* is especially pertinent to comparisons between the TMB-high and -low subsets, as non-oncogenic passenger mutations are more likely to occur in the TMB-high subset than the TMB-low subset. By reporting pathogenic variants specifically, our dataset is more likely to represent the clinically relevant phenotype. In our analysis, *KEAP1* mutations were identified in almost twice the proportion of patients with TMB-high than TMB-low tumors (**Figure 1B**). This difference between patients with TMB-high and TMB-low status was also apparent in the subgroups with either PD-L1-positive or PD-L1 negative tumors (**Figure 1C**), and in the non-squamous but not the squamous subgroup (**Supplementary Figure S1A**).

The rate of *STK11* mutations (occurring alone and concurrently with *KRAS* mutations) was substantially higher in the subset with PD-L1 negative tumors, almost twice the rate in the subset with PD-L1 positive tumors (**Figure 1D**). This difference between patients with PD-L1 negative and PD-L1 positive tumors was also apparent in the subgroups with either TMB-high or TMB-low status (**Figure 1C**) and in the non-squamous but not the squamous subgroup (**Supplementary Figure S1B**).

## Perspectives on the use of *STK11* and *KEAP1* biomarker testing in current clinical practice

Currently, biomarker testing is routinely used at diagnosis of NSCLC, to determine the patient’s eligibility for one of the currently available targeted therapies. When feasible, testing for additional markers (other than actionable abnormalities) could provide more detailed and specific insights into the molecular pathology that drives the disease in individual patients. We believe that integrating *STK11* and *KEAP1* biomarker testing into routine practice can be valuable for clinicians making treatment decisions for patients with mNSCLC. Understanding the potential impact of these biomarkers on prognosis provides some foresight into how the patient’s disease would be likely to develop during planned treatment. Thus, broader genomic testing may lead to improvements in selection and sequencing of treatment.

At the Sarah Cannon Research Institute, Nashville, a 65-year-old male presented to MJ with mNSCLC and several metastatic brain lesions. In line with our usual biomarker testing practice, next-generation sequencing (NGS) and immunohistochemistry testing for PD-L1 in tissue specimens were performed. The patient’s tumor had an elevated PD-L1 expression of 75%, together with mutations in both *STK11* and *KRAS* G12D. TMB reported as part of NGS was 12 mut/Mb. The patient was treated with whole-brain radiation and an initial course of steroids, followed by pembrolizumab 200 mg every 3 weeks. Although his PS was robust at diagnosis, by the time the course of radiotherapy had been completed, he began to show signs of disease progression and systemic decline. We expected his condition to improve once IO was started, but unfortunately, he continued to weaken, until he became too frail to receive additional therapy. Nowadays, we would still use radiotherapy to treat this type of patient, as this can achieve clinically valuable improvements when added to CTx or IO. However, knowing the negative prognostic impact of *STK11* and *KEAP1* mutations, we would also consider combining radiotherapy with concurrent or sequential CIT.

Obtaining information on *STK11* and *KEAP1* mutation status ‘up-front’ may further inform the choice of therapeutic regimen offered to our patients. Given that the incorporation of an additional IO agent can impose a greater financial burden on patients than PD-(L)1 inhibition alone, and that many oncologists are cautious of immune-mediated adverse events (IMAEs) that may arise with dual ICIs, being able to select patients who are characterized by good response may help us to refine our treatment strategy.

At UC San Diego (UCSD) Health, a patient with a history of moderate smoking presented to SPP with non-squamous mNSCLC. Plasma was sent to a vendor for cell-free DNA (cfDNA) NGS testing, given minimal tissue availability and the need for rapid diagnosis. The patient was found to have a *KRAS* G12V mutation with co-mutations in *STK11* and *KEAP1.* The PD-L1 expression score was 0 and TMB was approximately 9 mut/Mb. As is typical for patients with these disease characteristics treated at UCSD Health, the patient began combination treatment with CTx, a PD-1 inhibitor, and an anti-CTLA-4 agent. The patient tolerated treatment well and remains in remission over a year into their therapy.

In our experience, IMAEs in patients receiving an additional CTLA-4 inhibitor with CIT can be managed by the tumor board, and this can prolong the benefits achieved with IO. In this case, the patient had an IMAE (Grade 2 immune colitis with no blood). This was managed by a tumor board which included an immunologist who recommended treatment with steroids. The patient responded rapidly to prednisone (1 mg/kg oral starting dose), and the dose was tapered over four weeks. When immune colitis recurred, treatment with vedolizumab (a selective biologic drug that decreases gut inflammation, with only limited system-wide immunosuppression) was initiated. CIT was continued successfully without the CTLA-4 agent for two years.

## Further considerations on biomarker testing

Comprehensive genomic profiling (CGP) is recommended by ASCO, ESMO, and NCCN Guidelines, and is specified in the College of American Pathologists, International Association for the Study of Lung Cancer, and Association for Molecular Pathology guidelines (8, 9, 60, 61). Guidelines suggest broad biomarker testing may be used to support access to new treatments or clinical trials (8). Most often, CGP is carried out using an NGS-based platform; many commercially-available assays (**Supplementary Table S1**) include *STK11* and *KEAP1* mutations in their gene panel. Although tissue sampling remains the gold-standard for diagnostic testing of mNSCLC, in our experience, plasma (‘liquid biopsies’) may also be suitable for analysis of *STK11* and *KEAP1* mutations, particularly for patients with advanced disease, where the rate of detection tends to be higher. In the case of tissue testing, the need to screen for an increasingly broad range of markers will require a sufficient quantity of high-quality specimens that may not be available to clinicians in some clinical settings (62). Furthermore, as patients with *STK11* and *KEAP1* mutations often show rapid clinical deterioration, there is a limited therapeutic window. Consequently, the relatively long turnaround time required for tissue biopsy and testing may prove to be unfeasible in some circumstances.

## Conclusions

We believe that all patients with NSCLC should receive NGS-based CGP at diagnosis, using tumor tissue or cfDNA (particularly when tissue is limited or unavailable). As shown in our analysis, the distinct patterns of mutational prevalence between PD-L1 and TMB subgroups only further highlight the complex relationship between these two biomarkers. In light of recent guidelines that TMB should not be used as a sole indicator for ICIs (63), there is a need to identify new confounding factors that may influence treatment outcomes. This could include improving TMB scoring through more robust filtering of ancestral bias, assessment of the mutational status of *KEAP1* and *STK11*, and integrating PD-L1 positivity into a comprehensive score predictive of response. Emerging evidence on the prevalence of these mutations in specific racial/ethnic groups may also help to shed light on the heterogeneous nature of such tumors, and may allow us to tailor our clinical practice accordingly (51).

Given their poor prognosis when receiving standard CIT, patients with *STK11* and *KEAP1* mutations should be offered clinical trials with novel agents that specifically target on these genomic mechanisms. Increasing the ease of NGS testing for all patients with NSCLC, including in the first-line setting, will be vital for identifying these patients before they initiate less effective treatments. Future prospective studies will have a critical role in evaluating *STK11* and *KEAP1* mutations as predictors of resistance to anti-PD-1 (only)-directed strategies, and in determining the efficacy of combinatorial strategies (including combinations with anti-CTLA-4). Other emerging biomarkers of potential interest include *TP53* and *SMARCA4*, both being associated with aggressive disease biology (64–67), and *LRP1B*, which is potentially predictive of favorable outcomes with IO (68, 69).

## Data Availability

The original contributions presented in the study are included in the article/**Supplementary Material**. Further inquiries can be directed to the corresponding author.
