# Supplementary material for: Clinical perspectives on the value of testing for STK11 and KEAP1 mutations in advanced NSCLC
Source: Front Oncol. 2024 Dec 5;14:1459737. doi: 10.3389/fonc.2024.1459737 (PMC11655323; doi:10.3389/fonc.2024.1459737)
Supplement: Supplementary file 1 [file DataSheet1.docx]

**Supplementary figures and tables**

**Figure S1. Incidence of *KRAS*, *STK11*, and *KEAP1* (co-)mutations by TMB^a^ status and histological subtype (A), and PD-L1 expression^b^ status and histological subtype (B)**

**A

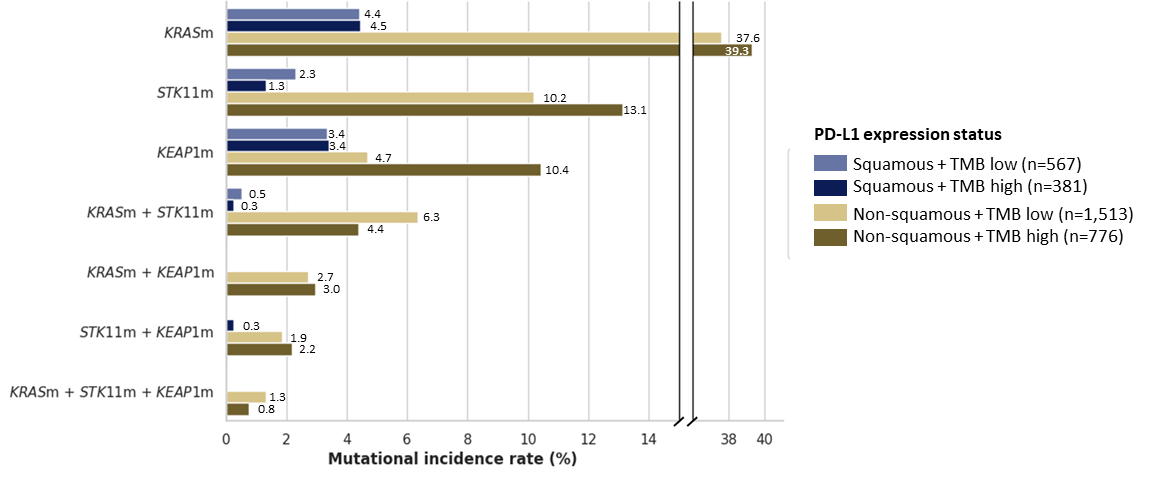
**

**B**

^
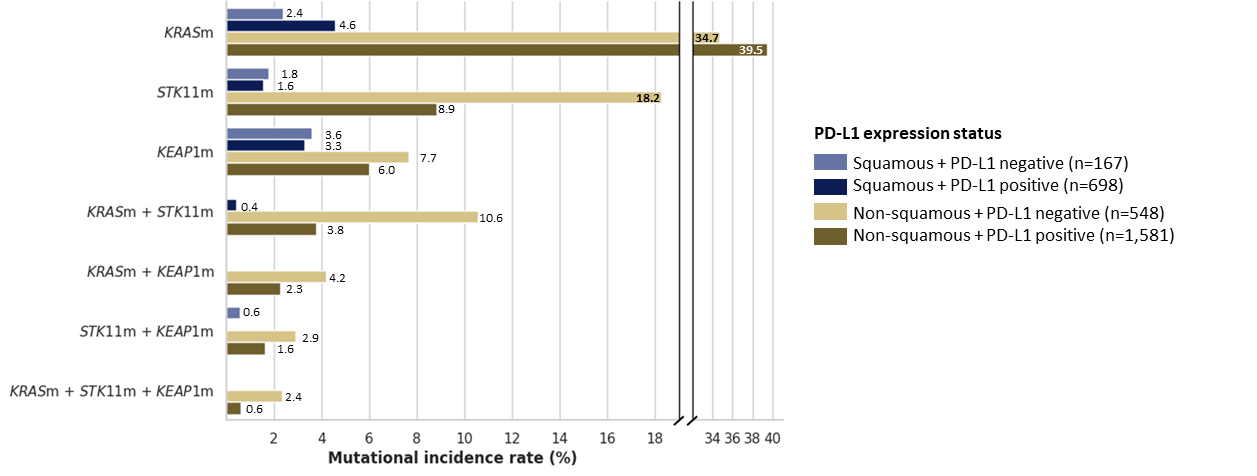
a^The PGDx elio™ tissue complete assay was used to determine TMB high (≥16.0 mut/Mb) vs. TMB low (<16.0 mut/Mb) status; ^b^The PD-L1 IHC 22C3 pharmDx assay was used to determine PD-L1 positive (TPS ≥1%) vs. PD-L1 negative (TPS <1%) status**.**

KEAP1(m), Kelch-like ECH-associated protein 1 (mutation-positive); KRAS(m), Kirsten rat sarcoma virus (mutation-positive); PD-L1, programmed cell death ligand-1; STK11(m), serine/threonine kinase 11 (mutation-positive); TMB, tumor mutational burden; TPS, tumor proportion score

**Table S1. Commercially available comprehensive genome profiling tests**

| Biomarker test | Sample type | Testing method | *KRAS* | *KEAP1* | *STK11* | Guideline-recommended biomarkers*^,1^ |
| --- | --- | --- | --- | --- | --- | --- |
| FoundationOne^®^CDx^2^ | Tissue | NGS/MPS | ✔ | ✔ | ✔ | ✔ |
| FoundationOne^®^Liquid CDx^3^ | Plasma | NGS/MPS | ✔ | ✔ | ✔ | ✔ |
| Guardant360^®4^ | Tissue | NGS | ✔ | ✔ | ✔ | ✔ |
| Ion Torrent™ Oncomine™ Dx Express Test^5^ | Tissue | NGS | ✔ | ✔ | ✔ | ✔ |
| oncoReveal™ Solid Tumor v2 Panel^6^ | Tissue or plasma | NGS | ✔ | ✔ | ✔ | ✔ |
| Resolution ctDx-Lung™ Assay^7^ | Blood | NGS (DNA) | ✔ | ✔ | ✔ | ✔ |
| Tempus xF assay^8^ | Blood | NGS (DNA) | ✔ | ✔ | ✔ | ✔ |
| LiquidHALLMARK^®9^ | Blood | NGS | ✔ | ✔ | ✔ | ✔ |
| Tempus xT assay^10^ | Tissue | NGS (DNA, RNA) | ✔ | ✔ | ✔ | ✔ |
| Molecular Intelligence^®^ Comprehensive Tumor Profiling^11^ | Tissue | NGS (DNA and RNA), IHC, ISH | ✔ | ✔ | ✔ | ✔ |
| OmniSeq INSIGHT^®12^ | Tissue | NGS (DNA and RNA) | ✔ | ✔ | ✔ | ✔ |
| NeoTYPE^®^ Lung Tumor Profile^13^ | Tissue | NGS, FISH, IHC, and other molecular methods | ✔ | ✔ | ✔ | ✔ |
| Endeavor^14^ | Tissue | NGS/MPS IHC (for PD-L1 expression) | ✔ | ✔ | ✔ | ✔ |

*NCCN-recommended biomarkers: *EGFR* mutation, *ALK* rearrangement, *KRAS* G12C mutation, *ROS1* rearrangement, *BRAF* V600E mutation, *NTRK1*/*2*/*3* gene fusion, *MET* exon 14 skipping mutation, *RET* rearrangement, *ERBB2* (*HER2*) mutation^1^

ALK, anaplastic lymphoma kinase; EGFR, epidermal growth factor receptor; FISH, fluorescent in situ hybridization; IHC, immunohistochemistry; ISH, in-situ hybridization; KEAP1, Kelch-like ECH-associated protein 1; KRAS, Kirsten rat sarcoma virus; MPS, massively parallel sequencing; NCCN, National Comprehensive Cancer Network; NGS, next-generation sequencing; PD-L1, programmed cell death ligand-1; STK11, serine/threonine kinase 11

**References**

1. Referenced with permission from the NCCN Clinical Practice Guidelines in Oncology (NCCN Guidelines^®^) for Non-Small Cell Lung Cancer V.11.2024. ^©^ National Comprehensive Cancer Network, Inc. 2024. All rights reserved. Accessed October 15, 2024. To view the most recent and complete version of the guideline, go online to NCCN.org. NCCN makes no warranties of any kind whatsoever regarding their content, use or application and disclaims any responsibility for their application or use in any way.
2. Foundation Medicine, Inc. FoundationOne^®^CDx (2024). https://www.foundationmedicine.com/test/foundationone-cdx [Accessed November 27, 2024].
3. Foundation Medicine, Inc. FoundationOne^®^Liquid CDx (2024). https://www.foundationmedicine.com/test/foundationone-liquid-cdx [Accessed November 27, 2024].
4. Guardant Health, Inc. Guardant360^®^ (2024). https://www.guardantcomplete.com/products/guardant360 [Accessed November 27, 2024].
5. Thermo Fisher Scientific, Inc. Oncomine Dx Express Test (2024). https://www.thermofisher.com/uk/en/home/clinical/diagnostic-testing/condition-disease-diagnostics/oncology-diagnostics/oncomine-dx-express-test.html [Accessed November 27, 2024].
6. Pillar Biosciences Inc. oncoReveal Solid Tumor Panel (2024). https://www.pillarbiosci.com/products/oncoreveal-solid-tumor-panel/ [Accessed November 27, 2024].
7. Laboratory Corporation of America^®^ Holdings. Resolution ctDx-Lung™ (2024). https://oncology.labcorp.com/ctDxLung [Accessed November 27, 2024].
8. Tempus AI, Inc. Tempus xF/xF+ Liquid Biopsy (2024). https://www.tempus.com/oncology/genomic-profiling/xf/?srsltid=AfmBOoobjoNSwKJuXW9EJWNVX94SuWF1XbhMgx-iyOXiwh2ypWwLb1zi [Accessed November 27, 2024].
9. Lucence Health Inc. LiquidHALLMARK^®^ (2024). https://www.lucence.com/liquid-biopsy/ [Accessed November 27, 2024].
10. Tempus AI, Inc. Tempus xT CDx Assay (2024). https://www.tempus.com/life-sciences/xt-cdx/?srsltid=AfmBOoqMS4Ab1NU9CGjL9uz6k7q8lMLzYZ7rB78jbZ3oqaPbdQ3trxy7 [Accessed November 27, 2024].
11. Caris Life Sciences. Testing Menu. https://www.carislifesciences.com/products-and-services/molecular-profiling/testing-menu/ [Accessed November 27, 2024].
12. Laboratory Corporation of America^®^ Holdings. OmniSeq INSIGHT (2024). https://oncology.labcorp.com/cancer-care-team/test-menu/omniseq-insight [Accessed November 27, 2024].
13. NeoGenomics Laboratories. NeoTYPE^®^ Lung Tumor Profile (2024). https://neogenomics.com/test-menu/neotyper-lung-tumor-profile [Accessed November 27, 2024].
14. National Library of Medicine. Genetic Testing Registry, Endeavor (2022). https://www.ncbi.nlm.nih.gov/gtr/tests/593438/ [Accessed November 27, 2024].
